# Genetic association study between TAB2 polymorphisms and noise-induced-hearing-loss in a Han Chinese population

**DOI:** 10.1371/journal.pone.0251090

**Published:** 2021-05-11

**Authors:** Guangzhi Yang, Boshen Wang, Dawei Sun, Huimin Wang, Mengyao Chen, Hao Chen, Baoli Zhu

**Affiliations:** 1 School of Public Health, Southeast University, Nanjing, Jiangsu, China; 2 Department of Prevention and Control for Occupational Disease, Jiangsu Provincial Center for Disease Prevention and Control, Nanjing, Jiangsu, China; 3 School of Public Health, Nanjing Medical University, Nanjing, Jiangsu, China; Shanghai Jiao Tong University, CHINA

## Abstract

Noise-induced-hearing-loss(NIHL) is a common occupational disease caused by various environmental and biological factors. To investigate the association between TAB2 and the susceptibility of NIHL of people exposed to occupational environments, a genetic association study was performed on selected companies with 588 cases and 537 healthy control subjects. Five selected single nucleotide polymorphisms (SNPs) in TAB2,incoluding rs2744434, rs521845, rs652921, rs7896, rs9485372, were genotyped after a collection of DNA samples. Evident differences in participants between the case group and the control group reveals the result that people with the TAB2 has a high probability of getting NIHL. The results show that rs521845 is deeply associated with the risk of NIHL and is available for the diagnosis in the future.

## Introduction

Noise-induced-hearing-loss(NIHL) is classified as a form of sensorineural hearing deficits and a major contributor to occupational disease burden in the world [1]. Patients with NIHL are reported to have a higher possibility getting depressive symptoms [2]. It has been evaluated that almost one-third of all cases can be ascribed to noise exposure and over 12% of the citizens worldwide is in danger of hearing loss from noise [3]. Even in America, NIHL ranks second among the most common occupational diseases [4]. NIHL is well acknowledged to be associated with occupational environment and genetic inheritance. Unhealthy lifestyles may also contribute to the incidence of NIHL [5–7]. Varieties of occupational workers and students have noise exposure and are likely to suffer from NIHL [8–10]. Due to its extensive influence and detriment, more scientists pay attention to its therapy. It is well established that individual susceptibility is one of three characteristics of NIHL [11]. It is no wonder that many researchers spend lots of effort on genetic polymorphisms of hearing loss and doctors place an emphasis on the genetic therapy of NIHL [12]. As gene-based therapies have made progress in medicine, it is encouraged to conduct a related study associated with TAB2 and hearing loss [13, 14]. Besides, researching on genetic polymorphisms is favorable to construct genetic risk prediction models in the aimed population [15].

TAB2 functions as an important component of inflammasome that has been implicated in inflammation. And haploinsufficiency of TAB2 was blamed for a variety of congenital heart defects [16]. In the immune system, TAB2 works as an important intermediate in the IMD signaling pathway and take on a great responsibility for the innate response in response to bacterial and viral infection [17]. TAB2 is also indispensable for B cell activation leading to Ag-specific Ab responses [18]. Additionally, TAB2 gene polymorphism was associated with Epithelial ovarian cancer susceptibility [19].

The association between genetic variants exist in DNMT1, DNMT3A, KCNQ4, GRM7 genes in Chinese Han(CHB) and susceptibility to NIHL were explored previously [20–22]. A majority of evidence indicate that TAB2 plays a critical role in congenital heart defects and epithelial ovarian cancer; however, there are no studies conducting the relationship between TAB2 and NIHL. Genotypes related to NIHL in some SNPs were significant, and it suggests that genotyping these SNPs is a prerequisite to survey the illness relations. Accordingly, a case-control study was implemented to explicate associations between TAB2 and individual susceptibility of NIHL.

## Material and methods

### Subjects

Staffs who obtained medical examinations conducted in the target industry companies of Jiangsu Province were selected as the subjects in this study. These participants were included on account of their steady occupational environment and their long time noise exposure. These workers were divided by several groups and were arranged in different workdays so that the results of examination can be accurate. As a result, 1,125 individuals took part in this medical routine checkup, which include electrocardiograph, urine routine test, blood routine test and some other auxiliary examinations. The hearing ability of these participants was judged by pure-tone audiometry (PTA). After the examination, each individual subject was presented a questionnaire by professional interviewers to collect their private living habits and other basic medical information. Participants with innate or acquired diseases that could influence normal hearing ability or those who have taken drugs with ototoxicity were excluded from this study.

Based on the principle of informed consent, the written agreement was signed by each participant. And this investigation was approved by the Institutional Review Board of Jiangsu Provincial Centre for Disease Prevention and Control.

### Assessment of audiometry and noise exposure

Sound pressure individual noise meters (Noise-Pro, Quest, USA) was adopted in different working areas to evaluate noise exposure intensity every four months. Each subject was required to be away from the noise exposure for more than 12 hours anterior to hearing test. PTA was conducted by excellent specialist in a sound-proof chamber with the assistance of a Madsen Voyager 522 audiometer (Kastrup, Denmark) at the speech frequencies (500, 1000, 2000 Hz) and high frequencies (3000, 4000, and 6000 Hz).

On the basis of the standards of ISO 2013: 1999, the definitions of normal hearing ability and hearing loss were established. To eliminate the confounding effects, the collected data of subjects were regulated by basic demographic information on the basis of the standards of occupational NIHL(GBZ49-2007).

### Group principles of NIHL and control participants

The occupational noise exposure strength and time of all participants exceed 85dB and 8 hours respectively, which conforms to the inclusion criteria of noise exposure. Binaural hearing limits of these workers at high frequencies transcend 25 dB(A) were established as NIHL participants of this research. Similarly, the binaural hearing limits of these industry workers at high frequencies inferior to 25 dB(A) were established as normal hearing participants.

The method of frequency matching between NIHL cases and controls was carried out to rule out influences of other variables except gene polymorphisms. Age, gender, occupational harmful factors and occupational noise working years were chosen as matching factors in this analysis. Ultimately, an aggregate number of 1125 participants (588 cases-537 controls) were included in this study.

### Selection and genotyping of target SNPs

By analyzing the previous articles in genetics online, the selection of target SNPs in TAB2 was conducted. Establishment of the possibly functional polymorphisms was done to satisfy the following criteria: the value of MAF in CHB exceeded 0.10; situated in areas except non-coding regions; r2 adopted for LD exceeded 0.8. Based on these requirements, an aggregate number of 13 SNPs were selected eventually. By conducting a search on these selected SNPs in PubMed, five representative SNPs (rs2744434, rs521845, rs652921, rs7896, rs9485372) in previous research were selected for genotyping and subsequent research.

To acquire the DNA extraction of each subject, their peripheral blood specimens were collected subjected to the strict aseptic principles while performing this health examination. By reference to the manufacturer’s instructions, genetic DNA were required to be segregated by the specific kits(Qiagen, Dusseldorf, Germany). All the specimens of these subjects were stored at a temperature of -80°in a special refrigerator for the following steps of this research.

Genotyping was conducted on these selected SNPs applying the ABI 7900HT Real-time PCR System (Applied Biosystems, Foster City, CA, USA). To ensure the preciseness, five control groups are organized in each plate. SDS 2.3 automated software was adopted to achieve the purpose of allelic discrimination subsequent to the process of amplification. The analysis of these results was performed by two professional researchers in a blind way. For the sake of repeating these experiments, this study selects 15 percent of these samples at random. And the result of the test is completely in conformity with former results.

### Statistical analysis

SAS 9.3 software is conducted by a professional statistician to perform statistical analysis. Frequencies were adopted as the statistical description of represent categorical variables and mean±standard deviation(SD) was the statistical method applied to describe normally distributed continuous variables. Chi-square test for the purpose of the goodness-of-fit was performed in the SNPs in the TAB2 among all the subjects in control group to analyze their Hardy-Weinberg equilibrium. Comparison of the differences in age, noise exposure intensity, smoking years, working years in occupational exposure and the results of hearing test was performed by Student’s t-test. Pearson’s χ2 test was conducted to estimate the difference in categorical variables between control group and case group. Adjust ORs and 95% CIs of all the selected genotypes were calculated, subsequent to set multiple logistic regression models and attenuated on the basis of the age, gender, alcohol consumption and other obvious confounders.

## Results

### Basic clinical information of participants

The characteristics and clinical information of 588 NIHL cases and 537 controls were described in Table 1. The mean of age, working years, noise exposure strength for NIHL group and control group were 40.43±7.34 years; 18.35±0.361 years; 87.56±7.776 dB and 40.70±7.63 years; 17.87±0.380 years; 88.10±7.546 dB respectively. The results demonstrated that statistically significant dissimilarity was not found between case group and control group in respect to the matched factors(age, gender, tobacco consumption, alcohol consumption, working years, noise exposure strength), which indicates that there is a well-matched relationship between the two groups. However, the average binaural hearing thresholds of NIHL patients in this examination at high frequency (38.30±13.39 dB) were conspicuously higher compared to control subjects (15.35±5.71 dB).

**Table 1:** Demographic characteristics of study subjects.

Variables	Cases (n = 588)		Controls (n = 537)		*P*
	n	%	n	%	
Age (years)					0.533
Mean±SD	40.43±7.34		40.70±7.63		
≤ 35	152	25.85	146	27.19	
35–45	290	49.32	242	45.07	
> 45	146	24.83	149	27.74	
Sex	SEX				0.791
Male	553	94.05	503	93.67	
Female	35	5.95	34	6.33	
Smoking					0.845
Now	333	56.63	296	55.12	
Ever	13	2.21	11	2.05	
Never	242	41.16	230	42.83	
Drinking					0.937
Now	246	41.84	221	41.15	
Ever	11	1.87	9	1.68	
Never	331	56.29	307	57.17	
Work time with noise (years)					0.362
Mean±SD	18.35±0.361		17.87±0.380		
≤ 16	268	45.58	266	49.53	
> 16	320	54.42	271	50.47	
Expose level with noise (dB)					
Mean±SD	87.56±7.776		88.10±7.546		0.482
≤ 85	202	42.08	169	38.58	
85–92	96	20.00	87	19.86	
> 92	182	37.92	182	41.56	
High frequency hearing threshold shift (dB)					
Mean±SD	38.30±13.39		15.35±5.71		<0.001
≤ 26	409	69.56	62	11.55	
> 26	179	30.44	475	88.45	

### Analysis of selected SNPs and its associations with the risks of NIHL

The elemental information of target SNPs and p-values for Hardy-Weinberg tests were presented in Table 2. The functions of rs2744434, rs521845, rs652921, rs7896 and rs9485372 are 3_prime_UTR_variant, ntron_variant, coding_sequence_variant, 3_prime_UTR_variant and intron_variant respectively. P-values of goodness-of-fit χ2 tests of rs521845, rs7896 and rs9485372 were more than 0.05, indicating that the three SNPs of control group conform to the requirements of HWE.

**Table 2:** General information of selected SNPs and Hardy-Weinberg test.

SNP	Alleles	Chromosome	Functional Consequence	MAF		*P* for HWE ^b^
				Control	Database	
rs2744434	T/C	6:149410768	genic_downstream_transcript_variant,3_prime_UTR_variant	0.466	0.458	0.017
rs521845	T/G	6:149350562	ntron_variant,genic_downstream_transcript_variant	0.425	0.36	0.108
rs652921	A/G	6:149409710	coding_sequence_variant,genic_downstream_transcript_variant, synonymous_varian	0.464	0.123	0.018
rs7896	C/G	6:149410340	genic_downstream_transcript_variant,3_prime_UTR_variant	0.08	0.195	0.898
rs9485372	A/G	6:149287738	genic_upstream_transcript_variant,genic_downstream_transcript_variant,intron_variant	0.416	0.173	0.978

Analysis of the genotypic distributions in NIHL cases and hearing controls with 5 genetic models were performed for the purpose of illustrating the associations between target SNPs and NIHL hazards as far as possible (Table 3).

**Table 3:** Distribution of five polymorphisms and the association with NIHL.

Genetic models	Genotypes	Cases		Controls		*P* ^a^	Adjusted OR
		*n* = 588	%	*n* = 537	%		(95% CI)^b^
rs2744434						ref	
Codominant	CC	121	20.58	139	25.88	0.097	1.00
	CT	322	54.76	270	50.28		0.696(0.514–0.941)
	TT	133	22.62	115	21.42		0.731(0.511–1.047)
Dominant	CC	121	20.58	139	25.88	0.031	1.00
	CT/TT	455	77.38	385	71.69		0.706(0.529–0.942)
Recessive	CC/CT	443	75.34	409	76.16	0.65	1.00
	TT	133	22.62	115	21.42		0.943(0.706–1.259)
Alleles	C	564	47.96	548	51.02	0.119	1.00
	T	588	50.00	500	46.55		1.489 [1.167~1.899]
rs521845							
Codominant	TT	166	28.23	152	28.31	0.039	1.00
	TG	302	51.36	252	46.93		0.895(0.675–1.187)
	GG	79	13.44	102	18.99		1.457(1.018–2.121)
Dominant	TT	166	28.23	152	28.31	0.042	1.00
	TG/GG	381	64.80	456	84.92		1.004(0.767–1.315)
Recessive	TT/TG	468	79.59	404	75.23	0.014	1.00
	GG	79	13.44	102	18.99		1.550(1.110–2.166)
Alleles	T	632	53.74	556	51.77	0.175	1.00
	G	460	39.12	456	42.46		0.894 [0.698~1.145]
rs652921							
Codominant	AA	133	22.62	114	21.23	0.127	1.00
	AG	323	54.93	270	50.28		0.961(0.710–1.302)
	GG	122	20.75	137	25.51		1.348(0.941–1.930)
Dominant	AA	133	22.62	114	21.23	0.654	1.00
	AG/GG	445	75.68	407	75.79		1.063(0.796–1.420)
Recessive	AA/AG	456	77.55	384	71.51	0.043	1.00
	GG	122	20.75	137	25.51		1.386(1.038–1.849)
Alleles	A	589	50.09	498	46.37	0.139	1.00
	G	567	48.21	544	50.65		1.073 [0.842~1.366]
rs7896							
Codominant	CC	503	85.54	452	84.17	0.758	1.00
	CG	79	13.44	80	14.90		1.161(0.820–1.643)
	GG	4	0.68	3	0.56		0.780(0.173–3.515)
Dominant	CC	503	85.54	452	84.17	0.525	1.00
	CG/GG	83	14.12	83	15.46		1.140(0.811–1.603)
Recessive	CC/CG	582	98.98	532	99.07	0.796	1.00
	GG	4	0.68	3	0.56		0.764(0.170–3.441)
Alleles	C	1085	92.26	984	91.62	0.586	1.00
	G	87	7.40	86	8.01		1.08 [0.688~1.696]
rs9485372							
Codominant	AA	102	17.35	96	17.88	0.937	1.00
	AG	277	47.11	255	47.49		0.970(0.694–1.357)
	GG	195	33.16	173	32.22		0.937(0.656–1.339)
Dominant	AA	102	17.35	96	17.88	0.813	1.00
	AG/GG	472	80.27	428	79.70		0.957(0.697–1.314)
Recessive	AA/AG	379	64.46	351	65.36	0.737	1.00
	GG	195	33.16	173	32.22		0.959(0.741–1.240)
Alleles	A	481	40.90	447	41.62	0.721	1.00
	G	667	56.72	601	55.96		0.872 [0.68~1.117]

A gene link analysis in respect to five SNPs between case group and control group in the current research was conducted. Great evidence supports that rs521845 presented the genotype frequency obviously differently between cases and controls (P = 0.039, 0.042 and 0.014 in three genetic models respectively). Subsequent to the adjustment of confounding factors with a logistic regression model, the distribution of genotype between two groups still stayed different. The risks for NIHL of participants carrying homozygous wild-type GG were higher than subjects carrying homozygous wild-type TT (OR = 1.457, 95% CI = 1.018–2.121) in the codominant model of rs521845. When it comes to the allelic model, a significant increase of NIHL risk in rs521845 variant G allele (OR = 0.894 [0.698~1.145]) was not observed. Therefore, the association between genetic variation in rs521845 of TAB2 gene and NIHL risk in Chinese Han population can be established based on our analysis.

### Association between NIHL susceptibility and various genotypes

**Fig 1:**
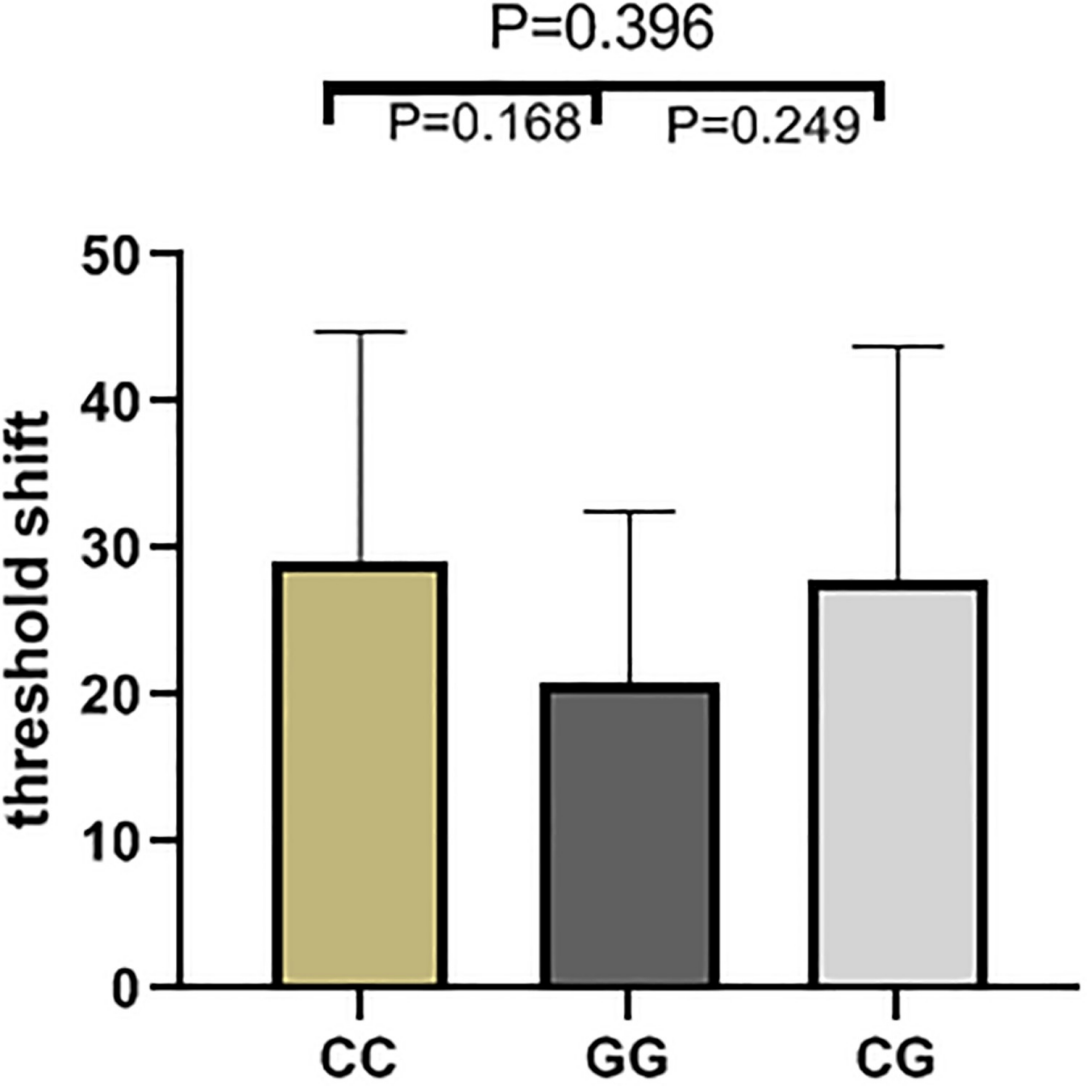
The genotypic distributions of rs2744434. The height of each column is the value of hearing threshold and each column stands for 1 genotype.

**Fig 2:**
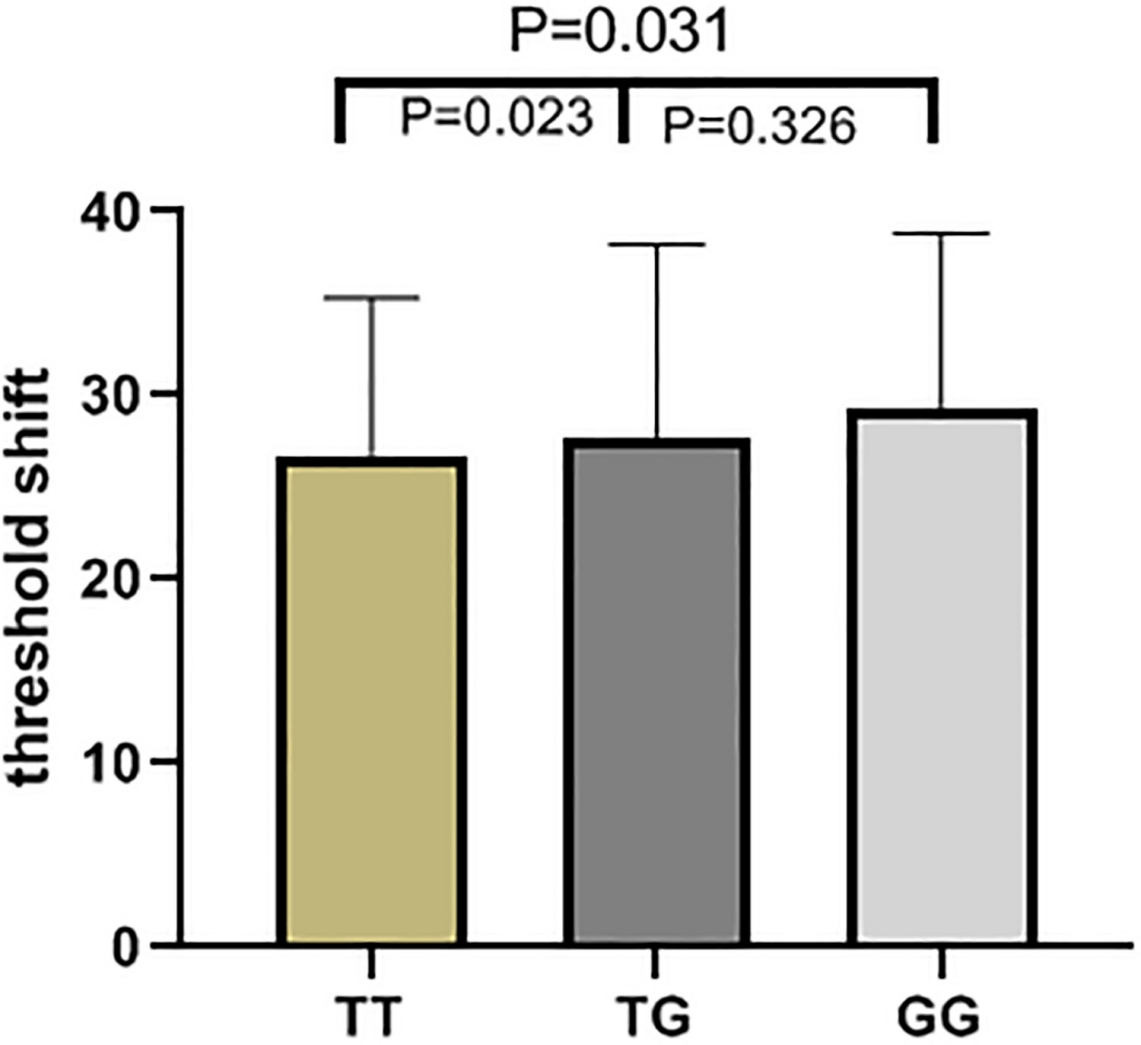
The genotypic distributions of rs521845. The height of each column is the value of hearing threshold and each column stands for 1 genotype.

**Fig 3:**
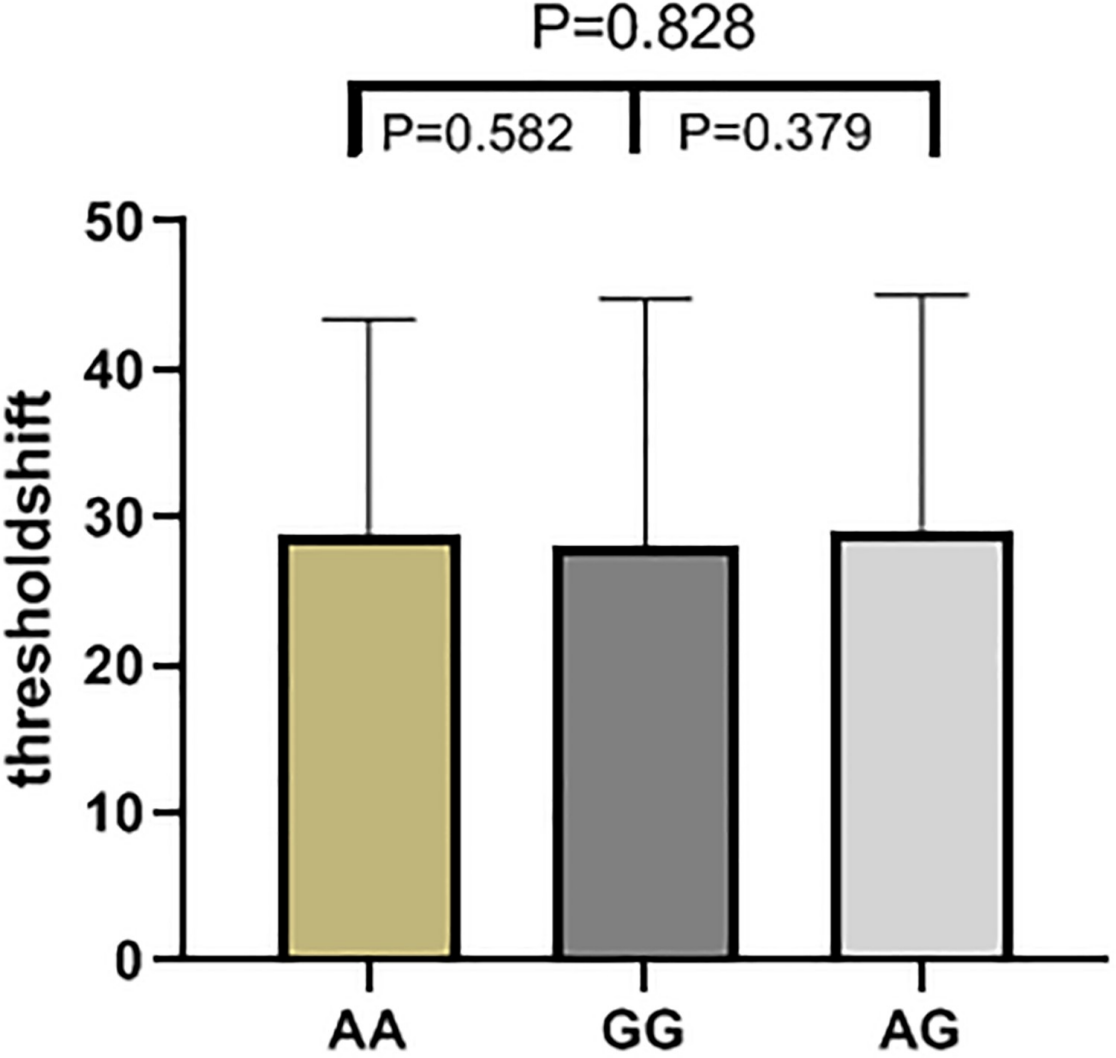
The genotypic distributions of rs652921. The height of each column is the value of hearing threshold and each column stands for 1 genotype.

**Fig 4:**
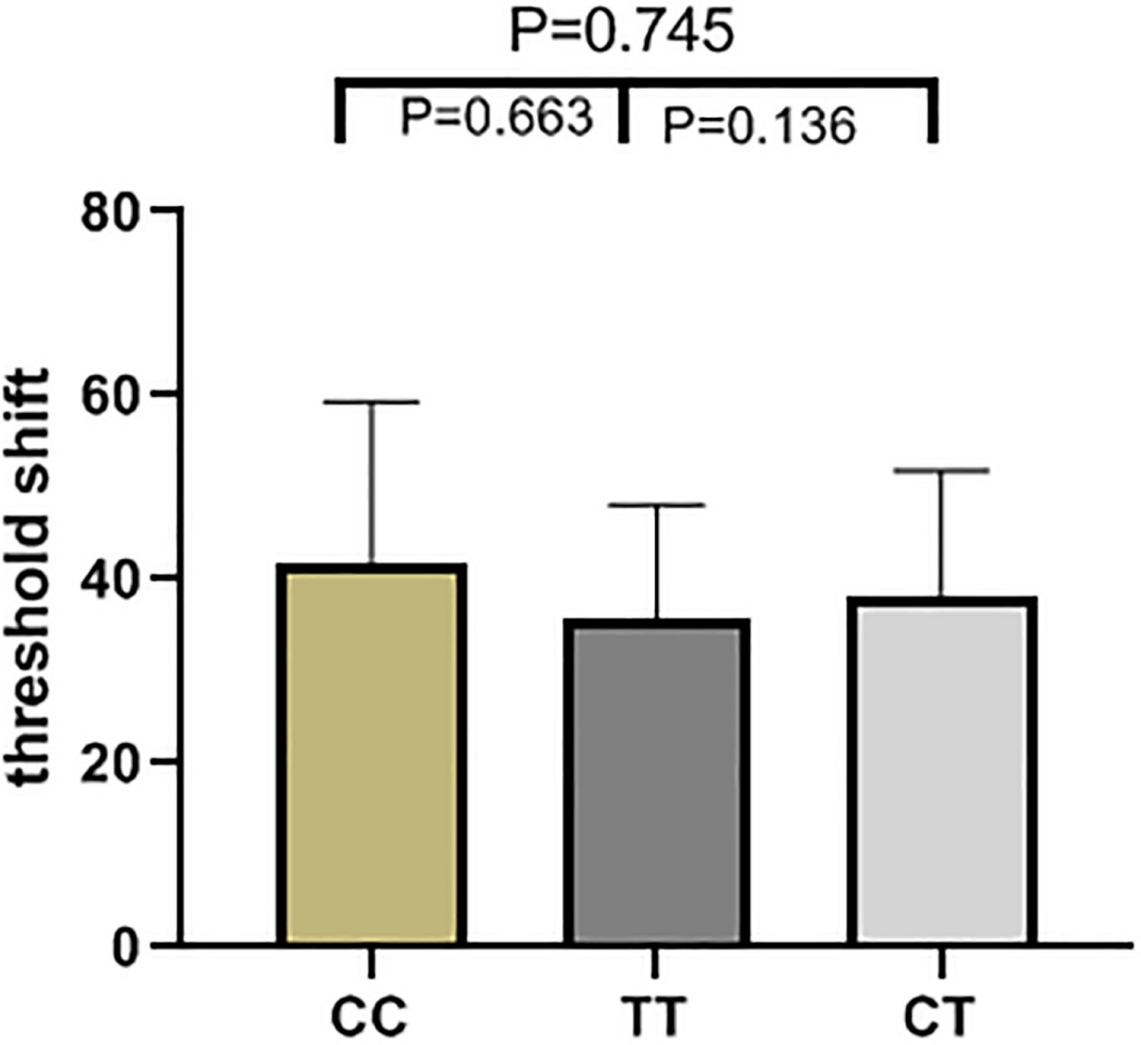
The genotypic distributions of rs7896. The height of each column is the value of hearing threshold and each column stands for 1 genotype.

**Fig 5:**
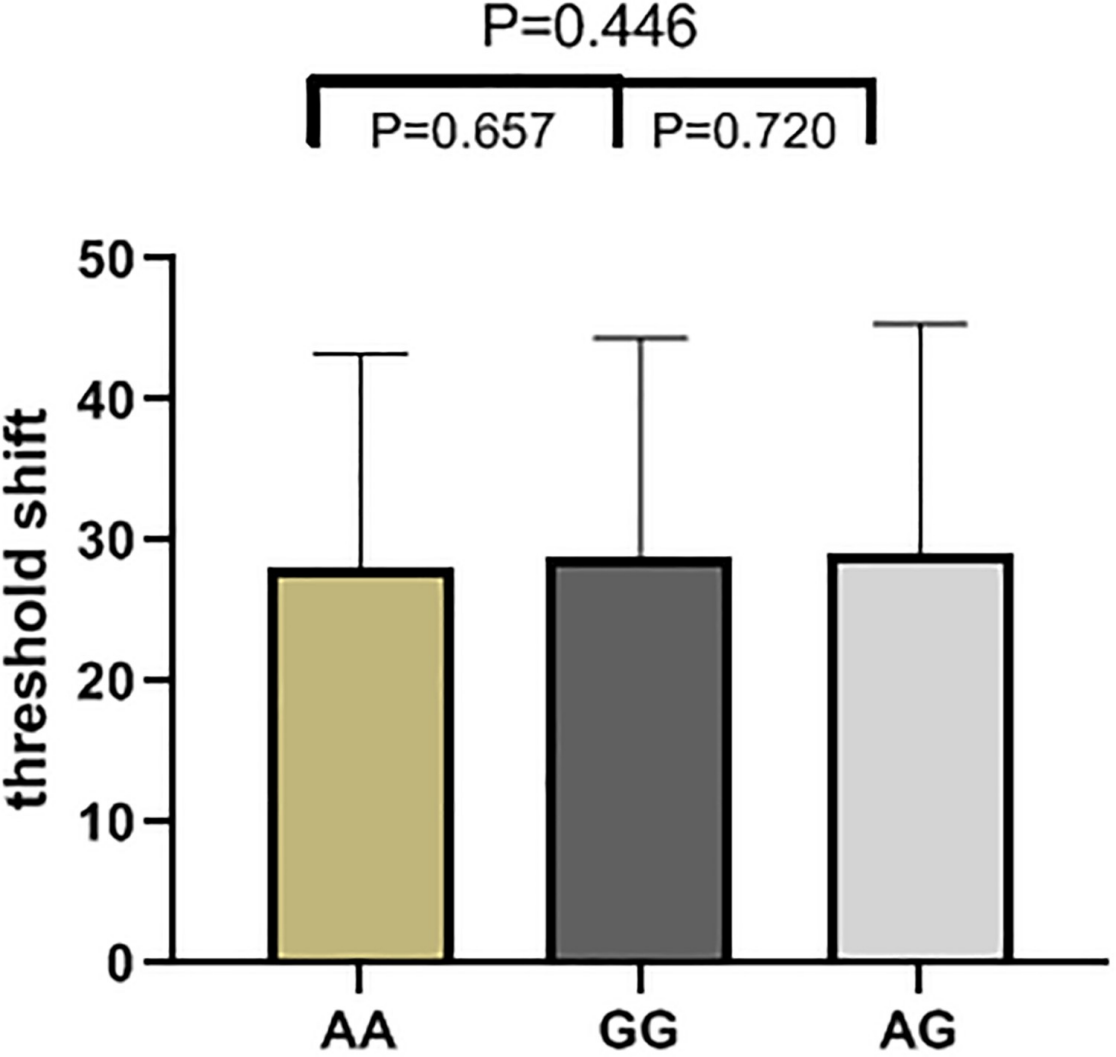
The genotypic distributions of rs9485372. The height of each column is the value of hearing threshold and each column stands for 1 genotype.

The results of high frequency hearing threshold of various genotypes in rs2744434, rs521845, rs652921, rs7896 and rs9485372 in 1125 study subjects were vividly described (Figs 1–5). Bases on the statistical analysis, the values of the threshold shifit of various genotypes in rs521845 were significantly different compared to other 4 SNPs. The difference of hearing threshold shift between subjects with rs521845 TG,GG genotype and subjects with TT genotype was obviously significant(P = 0.023, P = 0.031). It can be inferred that people with TG and GG genotype in rs521845 are at higher risks of NIHL than those with TT genotype. This research analyzed the distribution of genotypes and confirmed the association between TAB2 polymorphisms and NIHL susceptibility. Moreover, the function of genetic variations (G > T) in rs521845 to NIHL vulnerability was determined in this analysis.

### Distribution of inferred haplotypes in the subjects and their links with risk of NIHL

It is well acknowledged that the association between a haplotype and SNP alleles always exists. Generally, the identification of some important alleles of a target haplotype and statistical analysis of their association can be helpful, to some extent, to observe similar polymorphisms of other sites exist in this chromosome. The analysis of the five target SNPs in the aspect of haplotype frequencies between two groups was conducted (Table 4). Obvious protection effect was observed that the haplotype (rs2744434 C, rs521845 T, rs652921 A, rs7896 C and rs9485372 A) remarkably lowered the hazards of NIHL (OR = 0.488 0.346~0.687; P < 0.001) compared to others. The association between TAB2 polymorphisms and the increase of NIHL vulnerability was further confirmed to some degree by the results. This association research was of great significance for it placed an emphasis on the relation between rs521845; the haplotype, rs2744434 T, rs521845 G, rs652921 G, rs7896 G and rs9485372 G in the TAB2 gene and the vulnerability to NIHL in Chinese people. In conclusion, the association between the SNPs and the susceptibility to NIHL in long -time noise exposure workers was established based on our analysis.

**Table 4:** Distribution of inferred haplotypes in the subjects and their links with risk of NIHL.

Haplotypes	Case (*n* = 588)		Control (*n* = 537)		*P*	Adjusted OR	Global *P*
	n	%	n	%		(95% CI)	
TGGCG	126	26.2	150	30.7	0.051	0.76 [0.577~1]	<0.001
CGGCA	22	4.5	33	6.7	0.127	0.632 [0.363~1.099]	
CGGCG	38	7.9	23	4.7	0.066	1.648 [0.968~2.807]	
CTACA	60	12.5	107	21.9	<0.001	0.488 [0.346~0.687]	
CTACG	65	13.5	47	9.6	0.11	1.388 [0.934~2.063]	
TTACG	48	10	45	9.2	0.913	1.038 [0.678~1.589]	
TTACA	63	13.1	29	5.9	<0.001	2.253 [1.426~3.558]	

## Discussion

TAB2 is a gene that plays an important role in circulation system. TAB2 polymorphisms were associated with dilated cardiomyopathy susceptibility and prognosis in the Chinese population. Shen Can [23] proposed that C carriers (CT/CC) of rs652921 and G carriers (TG/GG) of rs521845 had a higher dilated cardiomyopathy risk and CC homozygote of rs652921 had a worse dilated cardiomyopathy prognosis. Permanyer [24] proposed that a variant in the TAB2 gene is associated with syndromic congenital heart disease, displaying congenital myxomatous degenerative heart valve disease, mild dysmorphic fascial anomalies and short stature in the surveyed family. Cheng’s findings [25] suggest that TAB2 haploinsufficiency is a risk factor for Hypoplastic left heart syndrome (HLHS) and expands the phenotypic spectrum of this microdeletion syndrome. Chromosomal single nucleotide polymorphism (SNP) microarray analysis and molecular testing for a TAB2 loss of function variant should be considered for individuals with HLHS, particularly in those with additional non-cardiac findings such as intrauterine growth retardation, short stature or dysmorphic facial features. TAB2 is an activator of MAP 3 K7/TAK1, which is required for the IL-1 induced signal pathway. Microdeletions encompassing TAB2 have been detected in various patients with congenital heart defects(CHD), indicating that haploinsufficiency of TAB2 causes CHD. To date, seven variants within TAB2 were reported associated with CHD, only two of them are nonsense mutations. And a novel TAB2 nonsense mutation is reported to be associated with CHD in a Chinese population by Chen [26]. Association between TAB2 polymorphisms and myocardial diseases were confirmed by many researchers, while no studies explored the association between gene polymorphisms and NIHL.

An association analysis on five target SNPs within TAB2 in 588 NIHL cases and 537 controls was conducted in the present study. The genotypic distributions of rs521845 in TAB2 gene between cases and controls was significantly different in statistics. Subsequent to the analysis of multivariate logistic regression, the correlation between rs521845 G and risk of NIHL was confirmed. Correspondingly, the hearing threshold shift of various genotypes in rs521845 in high frequency remained statistically different. Based on a lot of evidence, the effect of TAB2 polymorphisms on NIHL susceptibility in CHB can be determined.

Up to now, there are no articles depicting the association between TAB2 polymorphisms and NIHL. Samarajeewa [27] found that the downstream transcriptional response to Wnt activation partly underlies the regenerative capacity of the mammalian cochlea. TAB2 is a putative TAK1 interacting protein that is involved in the regulation of TAK1. TAB2 could directly interact with NLK and function as a scaffold protein to facilitate the interaction between TAK1 and NLK. And Li, M [28] found that TAK1-TAB2-NLK pathway may constitute a negative feedback mechanism for canonical Wnt signaling. From the two articles, we suspect that TAB2 may associate with NIHL. Based on the widely accepted opinion that it is sufficient to replicate an association study at the gene level rather than necessarily at the SNPs level, we specifically conducted this current research in a Chinese population with a large sample size to further confirm the association between TAB2 gene polymorphism and susceptibility to NIHL by analyzing five SNPs that have not been reported yet.

This analysis of gene-by-environment interaction on NIHL is credible for some researchers have conducted similar studies [29, 30]. The potential biological mechanism of this genetic variant on the disease development can be related to RNA modifications and immunodeficiency. RNA modifications can influence the expression results of genes. The role of N4-Acetylcytidine(ac4C) in RNA modifications were elucidated thoroughly recently by scientists [31]. As for its specific functions on RNA, ac4C is deeply associated with the translation level and stability of mRNA, the precision of translation ability of rRNA and translative protein fidelity of tRNA respectively [32, 33]. In the immune system, TAB2 plays a role on mediating in the IMD signaling pathway and takes on a great responsibility for the response to bacterial and viral infection [34]. Remarkably, rs521845 is located in synonymously coding region of TAB2, of which mutations may have a certain clinical significance. Mutations in synonymous codons are known to be linked with human diseases by affecting other features of protein biosynthesis rather than coding the amino acid sequence. Therefore, genetic variations in rs521845 within TAB2 can increase the risk of NIHL by influencing the process of RNA modification.

There are still some potential limitations in the current study. Firstly, the participants in this research were all Chinese published, therefore the trans-ethnic meta-analysis and subgroup meta-analysis according to ethnicity, sex, gene-dosage, and age, based on these five SNPs was not able to conducted for the limited genotype data [35, 36], so our results may likely be better generalized to Chinese Han and the extension to other ethnical populations is limited. Secondly, the clinical information of some subjects were not complete or accurate for some unexpected situations and could not be adopted properly. In addition, although the association between rs521845 in TAB2 and susceptibility to NIHL was significant, it is still difficult to use this variant to conduct early diagnosis under the framework of precision medicine [37], and in the future, a machine-learning model based on this genomic biomarkers to predict disease risk is warrant [38].

In conclusion, the association between rs521845 G allele of TAB2 and the hazards of NIHL is statistically significant and the finding is of great value to the early screening of NIHL in noise exposure workers. The functions of genetic polymorphism in TAB2 have an influence on both the incidence and the development of NIHL according to our conclusions. Nevertheless, additional analysis based on a larger sample size in different ethnical populations and the follow-up functional investigation on biological mechanism are warrant in the future.

## Supporting information

S1 File Questionnaire. (DOCX)
