# Supplementary material for: Genetic association study between TAB2 polymorphisms and noise-induced-hearing-loss in a Han Chinese population
Source: PLoS One. 2021 May 11;16(5):e0251090. doi: 10.1371/journal.pone.0251090 (PMC8112696; doi:10.1371/journal.pone.0251090)
Supplement: S1 File — (DOCX) [file pone.0251090.s001.docx]

**调查表**

问卷编号

**一、一般情况**

1.1 姓名 1.2 性别： 1=男 2=女

1.3 年龄 岁 1.4 工龄 年

1.5 身高 厘米 1.6体重 千克

1.7 工作岗位

1.10文化程度

**二、行为习惯**

2.1你是否吸烟？ 1=是 2=否

2.2你是否经常饮酒（每周至少饮两次）1=是 2=否

2.3工作之余，你是是否经常参加一些体育活动？ 1=是 2=否

2.4你是否觉得压力大？ 1=是，很大 2=是，一般 3=否

2.5你是否使用防护耳机？ 1=是 2=否

2.6你是否每天用耳机超过2小时？ 1=是 2=否

2.7你是否使用过耳毒性药物 1=是 2=否

**三、疾病史**

3.1 有无医生曾告诉你患有耳部感染 1=是 2=否

3.2 有无医生曾告诉你患有高血压 1=是 2=否

3.3 有无医生曾告诉你患有噪声性耳聋 1=是 2=否

3.4 有无动过中耳手术 1=是 2=否

3.4 有无耳部外伤史 1=是 2=否

**被调查者签名：**

**调查员：**

**日 期：**

**Questionnaire**

Questionnaire Number

General information

1.1 Name 1.2 Sex： 1=male 2=female

1.3 Age 1.4 Working year

1.5 Height 1.6 Weight

1.7 position

1.10 Education level

Regular habits

2.1Do you smoke 1=Yes 2=No

2.2Do you often drink（more than twice a week）1=Yes 2=No

2.3Do you excercise in your spare time 1=Yes 2=No

2.4Do you feel stressed 1=Yes 2=Yes,a little 2=No

2.5Do you use earplug 1=Yes 2=No

2.6Do you use earphone for over 2 hours daily 1=Yes 2=No

2.7Do you take ototoxic drugs 1=Yes 2=No

Medical history

3.1 Did you get ear infection 1=Yes 2=No

3.2 Did you get hypertension 1=Yes 2=No

3.3 Did you get noise-induced-hearing-loss 1=Yes 2=No

3.4 Did you have ear surgery 1=Yes 2=No

3.4 Did you have ear trauma 1=Yes 2=No

**Signature：**

**Investigator：**

**Date：**
